# Causal Association between Arm Fat, Left Leg Fat, and Trunk Fat Masses and Risk of Polycystic Ovarian Syndrome: A Mendelian Randomization Study

**DOI:** 10.2174/0113862073325251241101054306

**Published:** 2025-01-09

**Authors:** Yuhan Zhang, Wei Zhou, Qiong Su, Qi Chen

**Affiliations:** 1 Department of Obstetrics and Gynecology, The Second Affiliated Hospital, Jiangxi Medical College, Nanchang University, Nanchang, China;; 2 Jiangxi Key Laboratory of Molecular Medicine, The Second Affiliated Hospital, Jiangxi Medical College, Nanchang University, Nanchang, China;; 3 Center for Reproductive Medicine, Jiangxi Maternal and Child Health Hospital, Jiangxi Branch of the National Clinical Research Center for Obstetrics and Gynecology, Nanchang Medical College, Nanchang, China

**Keywords:** Polycystic ovarian syndrome, mendelian randomization, single nucleotide polymorphism, ovulatory dysfunction, prognosis, glucose tolerance

## Abstract

**Background:**

Observational studies have reported that arm fat, left leg fat, and trunk fat masses have different effects on polycystic ovarian syndrome (PCOS). However, the causal relationship between them remains unknown.

**Materials and Methods:**

A two-sample Mendelian randomization (MR) study was conducted by utilizing pooled data from the largest Genome-Wide Association Study (GWAS). Random effect inverse variance weighted (IVW) method, weighted median (WM), and MR-Egger regression analysis were the main statistical methods utilized. Finally, a sensitivity assessment was conducted. Cochran’s Q test was used to analyze heterogeneity, whereas MR-Egger regression (intercept term) was used to analyze horizontal pleiotropy. The leave-one-out analysis was performed to assess if MR estimates were impacted by a single nucleotide polymorphism (SNP) exhibiting significant horizontal pleiotropy.

**Results:**

This study discovered a significant positive correlation between left leg fat mass, arm fat mass, and trunk fat mass and genetic factors of PCOS (odds ratio (OR): 4.452, confidence interval (CI): 2.740−7.232, *p <* 0.001, OR: 3.321, CI: 2.248−4.907, *p <* 0.001, and OR: 2.518, CI: 1.722−3.682, *p <* 0.001, respectively).

**Conclusion:**

This study indicates that arm fat, left leg fat, and trunk fat masses may be genetically correlated with PCOS.

## 1 INTRODUCTION

*Polycystic* ovarian *syndrome* (PCOS), categorized as a heterogeneous *syndrome* of ovulatory dysfunction, *polycystic* ovarian morphology, and hyperandrogenism, *is* a highly prevalent endocrine and reproductive disorder [1]. Moreover, PCOS is frequently linked to excess weight, obesity [2-5], central obesity [6], dyslipidemia [7], insulin resistance [8], heightened oxidative stress [9], persistent low-level inflammation [10], heightened chances of developing metabolic syndrome [11], compromised glucose tolerance [12], type 2 diabetes [13], and potential cardiovascular diseases [14, 15]. The cause of PCOS is not fully understood. However, research indicates that PCOS is likely caused by a combination of genetic, environmental, and intrauterine factors [16-18]. Disease management and prognosis depend on a combination of interventions, including lifestyle adjustments (such as increased exercise, weight loss, and a balanced diet) [19, 20] and medications (such as antiandrogen drugs and metformin) [21-23], as well as ovulation induction therapy or assisted reproductive technologies for those with fertility issues. Women diagnosed with PCOS, particularly those who are not overweight, seem to have higher amounts of trunk [24-27], abdominal subcutaneous, and visceral fat [28-30] in comparison to controls matched for body mass index. Weight management is the primary treatment recommended for PCOS, and symptoms of PCOS typically improve when weight loss is between 5% and 10% [31]. Therefore, body composition is an important factor in the pathogenesis of PCOS. However, current clinical evidence is not sufficient to evaluate and manage PCOS, necessitating the need for key evidence-based knowledge.

Obesity is known to exacerbate insulin resistance and decrease metabolism in patients with PCOS. However, approximately 40–50% of patients with PCOS have a BMI within the normal range, so weight loss alone is not suitable for this population. Researchers have found that patients with PCOS, especially those who are non-obese, appear to have more trunk and visceral fat compared to the BMI-matched control group [32]. Therefore, studying the body fat distribution of PCOS patients is essential for understanding the relationship between PCOS and body fat deposition.

Mendelian randomization (MR) is an analysis of genetic variables that adheres to Mendelian inheritance principles. Utilizing data from genome-wide association studies (GWAS), this method employs single nucleotide polymorphisms (SNPs) as instrumental variables (IVs) to determine the potential causal connection between modifiable exposure and clinically significant outcomes [33-43]. It provides an effective approach to address the difficulties in conducting randomized controlled trials (RCTs) and the inability of observational studies to exclude confounding factors and reverse causal associations. Recently, Mendelian randomization has been widely used in medical research.

In summary, this study aimed to explore the causal relationship between PCOS and body fat distribution through comprehensive genetic analysis. The results showed that increased right arm fat mass, left leg fat mass, and increased trunk fat mass were significantly associated with the risk of PCOS. The findings of this study may serve as exposure factors in the development of PCOS.

## 2 MATERIALS AND METHODS

### 2.1 Study Design

This study used a two-sample MR randomized method to assess the causal association between right arm fat, left leg fat, and trunk fat masses and the risk of PCOS. All data were derived from the previously published GWAS. Since ethical consent and approval were already obtained in the original study, there was no need for any further ethical approval or informed consent. GWAS summary statistical data were retrieved, and SNPs that showed significant statistical correlation with arm fat, left leg fat, and trunk fat masses were extracted as genetic instrumental variables. GWAS summary statistical data related to PCOS outcomes were selected from the published large-scale GWAS meta-analyses, and gene outcome associations were extracted to conduct Mendelian randomization and the corresponding sensitivity analysis of the results.

### 2.2 Selection of Instrumental Variables

The exposure factor data obtained in this two-sample Mendelian randomization study were based on the Neale Lab study. Fat mass in the right arm was selected as the genetic instrumental variable of genome-wide significant SNPs (*P <* 5e-08) (ukb-a-283). The study included 331,226 participants. After removing the linkage imbalance (r2 ≤ 0.001), independent SNPs were selected, and the region width was set to 10000 kb to ensure each SNP was independent and excluded the influence of gene pleiotropy on the results. Furthermore, left leg fat mass was selected as the genetic instrumental variable of genome-wide significant SNPs (*P <* 5e-08) (ukb-a-279). The study included 331,275 participants who were predominantly of European descent. After removing the linkage imbalance (r2 ≤ 0.001), independent SNPs were selected, and the region width was set to 10000 kb to ensure each SNP was independent and excluded the influence of gene pleiotropy on the results. Finally, trunk fat mass was selected as the genetic instrumental variable of genome-wide significant SNPs (*P <* 5e-08) (ukb-a-291). The study included 330,995 participants of predominantly European ancestry. After removing the linkage imbalance (r2 ≤ 0.001), independent SNPs were selected, and the region width was set to 10000 kb to ensure each SNP was independent and excluded the influence of gene pleiotropy on the results.

### 2.3 Extraction of Outcome GWAS Data

The outcome GWAS dataset was selected from the Finnish GWAS database (finn-b-E4_POCS). In the FinnGen study, PCOS was defined using its corresponding international statistical classification of diseases (ICD) code. The study included 642 cases of PCOS and 118,228 controls.

### 2.4 Statistical Analysis

The foundation of reliable MR analysis rests on three key assumptions: the correlation hypothesis, the independence hypothesis, and the exclusivity hypothesis. To test the correlation hypothesis, the proportion of exposure variation R^2^ and F statistics that the instrumental variable can explain are calculated. If F > 10, the probability of violating the hypothesis and introducing weak instrumental variable bias is small. The other two hypotheses cannot be directly tested, but they can be evaluated by sensitivity analysis and other testing methods. All analyses were performed using the “MendelR” package in the R software (version 4.2.2). Three methods, InverseVarianceWeighted (IVW), WeightedMedian (WM), and MR-Egger regression analysis, were used for MR analysis. Finally, a sensitivity analysis was also carried out. Heterogeneity was tested using Cochran's Q statistics, while horizontal pleiotropy was tested using MR-Egger regression (intercept term). To determine if the MR estimate was influenced by an SNP with a significant horizontal pleiotropy effect, a leave-one-out analysis was performed.

## 3 RESULTS

### 3.1 Instrumental Variables

**Fig. (1):**
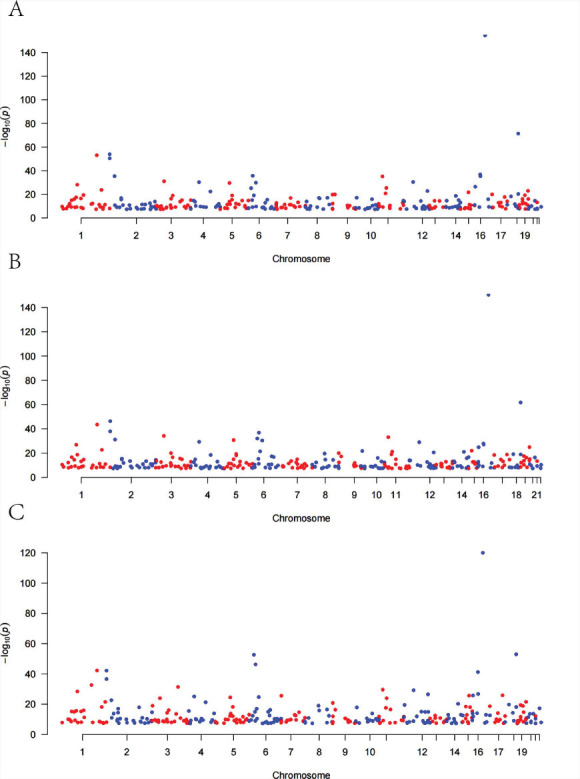
Manhattan plot of exposure factors. (**A-C**) Manhattan plot of right arm fat mass, left leg fat mass, and trunk fat mass corresponding to instrumental variables. In the Manhattan map, each data point represents a single nucleotide polymorphism (SNP), or variation, on the genome. The abscess represents the location of the genome, and the ordinate represents the negative logarithm of the *p*-value of the causal relationship associated with the exposed data. Data points are grouped and displayed according to different regions of the chromosome, while different colors usually indicate different chromosomes.

According to the screening criteria for instrumental variables in this study, 277 SNP instrumental variables were screened out for right arm fat mass (Fig. **1A**). The MR-Egger regression intercept terms were b = 1.49 and *p* = 0.011, respectively. A total of 291 SNP instrumental variables were selected for left leg fat mass (Fig. **1B**), with the MR-Egger regression intercept terms being b = 2.048 and *p* = 0.006, respectively. A total of 290 SNP instrumental variables were selected for trunk fat mass (Fig. **1C**), with the MR-Egger regression intercept terms being b = 0.953 and *p* = 0.11, respectively. In other words, there was no gene pleiotropy between the screened SNPs and the outcome, and SNPs were not directly related to the outcome. Therefore, the Mendelian randomization method was effective for causal inference in this study.

### 3.2 Mendelian Randomization Analysis

**Fig. (2):**
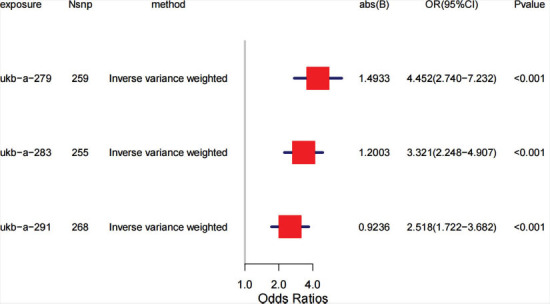
Odds ratios for three exposure factors and polycystic ovary syndrome. Risk profiles of three causal pairs (right arm fat mass, left leg fat mass, and trunk fat mass and PCOS) from Mendelian randomization analysis.

The regression results of the four models are presented in **Supplementary Tables 1-3**. As mentioned in the table, IVW results showed that genetically predicted right arm fat, left leg fat, and trunk fat masses had significant causal associations with the risk of PCOS. The higher the level of right arm fat, left leg fat, and trunk fat masses, the higher the risk of PCOS (Fig. **2**).

### 3.3 Heterogeneity Test

**Fig. (3):**
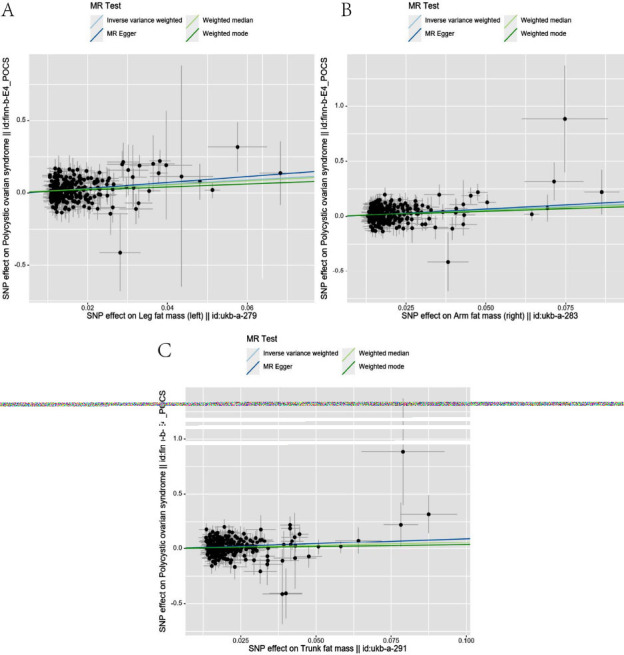
Scatter plots of causality. (**A-C**) Scatter plot of the causal relationship between right arm fat mass, left leg fat mass, and trunk fat mass.

**Fig. (4):**
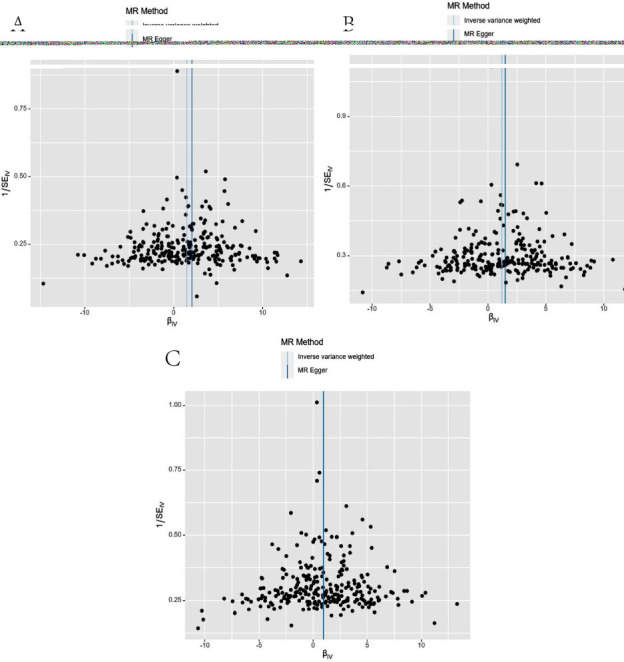
Funnel plot to assess heterogeneity. (**A-C**)Funnel plot of secondary MR analysis of right arm fat mass, left leg fat mass, and trunk fat mass. In this diagram, the width of the funnel represents the number of samples in different groups or categories, and the height of the funnel represents the distribution of the corresponding fat amount. The distribution of fat in different parts can be visually observed, and the heterogeneity between them can be compared.

The scatter plots show the effects of right arm fat, left leg fat, and trunk fat levels on the risk of PCOS (Fig. **3**). As presented in the funnel plot, all SNPs included were symmetrical, indicating that using SNPs as IV infers that the causal effect has minimal influence (Fig. **4**). The distribution of points in the funnel plot was roughly symmetrical, indicating the absence of an unbalanced directional horizontal pleiotropy.

### 3.4 Sensitivity Test

**Fig. (5):**
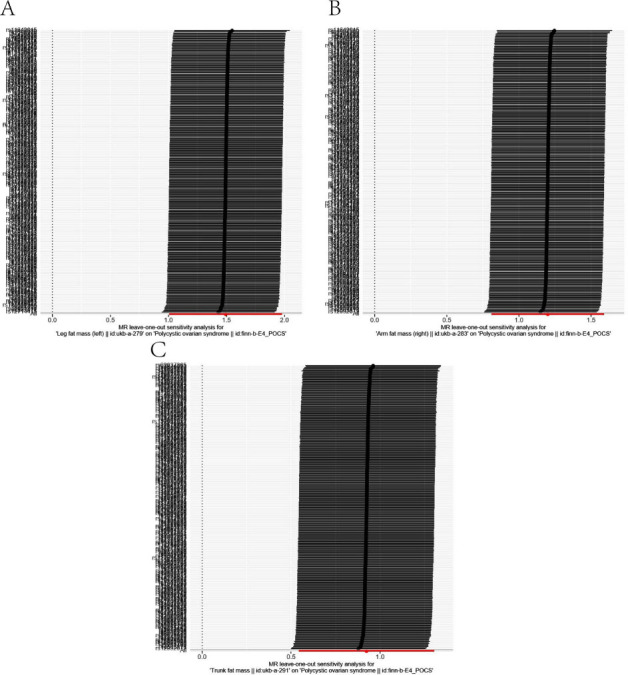
Leave-out analysis. (**A-C**) Forest Plot for leave-out tests of right arm fat mass, left leg fat mass, and trunk fat mass and the causal relationship between PCOS. In a forest map, each horizontal line represents an independent cause-and-effect relationship. Each square represents the effect size of the causal relationship, and the length of the square represents the range of its confidence interval. If the range of squares crosses the vertical zero-effect line (usually 1), then the causality is considered significant.

This study utilized a leave-one-out analysis for sensitivity analysis, and a forest map was constructed (Fig. **5**). The results showed that after removing any SNP, the remaining SNPs were all on the same side of the invalid line, implying that removing any SNP would not significantly impact the results, thereby highlighting the robustness of the MR results.

## 4 DISCUSSION

PCOS is a heterogeneous disease that involves many factors [1]. Due to factors, such as age, race, genotype [44-46], lifestyle [47-49], weight [50], and environmental factors [51], individual phenotypes vary greatly. However, many features of PCOS are reversible following lifestyle interventions, such as diet and exercise, and it has been found that pregnant women who follow a healthy lifestyle have a lower risk of complications [52]. As a consequence, non-pharmacological interventions are needed to limit obesity in the PCOS population. Some results provided evidence that reducing carbohydrate intake may be an ideal choice for women with PCOS. Reducing carbohydrate diet leads to a decrease in subcutaneous abdominal, intra-abdominal, and thigh intermuscular adipose tissue (-7.1%, -4.6%, and -11.5%, respectively) [53]. Once diagnosed with PCOS, it is recommended to develop a lifelong health plan that focuses on a healthy lifestyle, preventing excessive weight gain, optimizing fertility and pre-pregnancy risk factors, as well as preventing and treating various clinical features.

Patients with PCOS often exhibit an uneven distribution of body fat [54-56], which may lead to increased fat deposition in one arm or leg [57-59].

In addition, PCOS is often accompanied by insulin resistance, which promotes the accumulation of fat in the trunk area. Therefore, changes in body fat can be an external manifestation of hormone imbalance and metabolic abnormalities in patients with PCOS, reflecting the impact of this endocrine disease on body fat distribution. This change in fat distribution is not only one of the symptoms of PCOS but may also exacerbate other symptoms of the disease, such as irregular menstruation, infertility, and risk of diabetes. Through Mendelian randomization, this study concludes that the higher the fat content in the right arm, left leg, and trunk, the higher the risk of PCOS. Consistent with this finding, previous studies have reported that an increase in subcutaneous adipose tissue of the trunk is associated with insulin resistance and impaired glucose tolerance in women with PCOS [60]. However, the study also suggested that increased thickness of subcutaneous adipose tissue in the legs can prevent metabolic disorders in PCOS. Other studies have reported that women with higher leg fat have a higher efficiency in consuming dietary fatty acids, and their regulation is usually different from visceral fat [61]. As PCOS is a multi-phenotype disease, more evidence-based knowledge may be needed to support the relationship between leg fat and PCOS.

Currently, the majority of research suggests that hyperandrogenism, visceral obesity, and insulin resistance are the key clinical characteristics of PCOS [62-64]. Obesity can change the concentrations of various hormones, such as androgens, insulin, and hormone-like peptides known as adipocytokines. Androgens have the ability to trigger mild, persistent inflammation by enhancing the expression of androgen receptors in monocytes, which helps dysfunctional adipocytes release adipocytokines [65]. Patients with PCOS often have a certain degree of abdominal fat, which is strongly independent of insulin resistance and excess androgen [66, 67]. Compared to the general population matched with BMI, women with PCOS exhibited altered fat distribution and were more inclined to increased accumulation of visceral adipose tissue (VAT) [68], even within the normal range of BMI [69]. However, some studies have also confirmed that the fat distribution assessed by magnetic resonance imaging (MRI) and computed tomography (CT) in PCOS and the control group was similar, suggesting that central obesity may not be related to PCOS. This study found a correlation between trunk fat levels and PCOS. The results of this study will contribute to a reevaluation of the connection between PCOS and atypical fat distribution, as well as assist in the creation of tailored lifestyle interventions for individuals with PCOS. Given the etiological role of insulin resistance and the impact of obesity on hyperinsulinemia and androgen excess, a multidisciplinary lifestyle improvement that normalizes insulin resistance, improves androgen levels, and helps with weight management is considered a crucial preliminary treatment strategy. PCOS should be considered a high-risk disease during pregnancy, and there is currently a high level of dissatisfaction with the diagnosis and care of PCOS [70-74]. It is strongly recommended that women and medical personnel should increase their education and raise awareness, including providing high-quality, evidence-based resources.

Dysfunction of adipose tissue is closely related to metabolic disorders and plays an important role in the pathophysiology of polycystic ovary syndrome. Brown adipose tissue plays an important role in regulating overall body balance. Recently, in research, PCOS was inducted in female C57BL/6J mice by oral administration of 1mg/kg letrozole for 21 days. Then, the animals received BAT transplantation or were given BAT Exos, with healthy female mice serving as controls. The results showed that BAT Exos treatment significantly reduced the body weight of PCOS mice and improved insulin resistance. In addition, BAT exos improved ovulation function by reversing the non-periodicity of the estrous cycle, reducing circulating luteinizing hormone and testosterone, restoring ovarian function, and improving oocyte quality, resulting in higher pregnancy rates and litter size. These findings suggest that BAT Exos therapy may be a potential treatment strategy for PCOS, and the STAT3/GPX4 signaling pathway is an important therapeutic target for PCOS [75].

## 5 STUDY LIMITATIONS

This study poses certain limitations. Firstly, the relationship between PCOS and right arm fat, left leg fat, and trunk fat has not been elucidated from a deeper molecular mechanism. Furthermore, basic research is still required to prove the theoretical hypotheses.

## CONCLUSION

In summary, the findings from MR analysis suggest that arm fat mass, left leg fat mass, and trunk fat mass could potentially be linked to a higher likelihood of developing PCOS. The results of this research indicate that obesity could be a significant factor in the onset of PCOS, which could help uncover the reasons behind how obesity impacts the likelihood of developing PCOS.

## Data Availability

The ukb-a-28, ukb-a-279, and ukb-a-291 were retrieved from the previously published GWAS.

## LIST OF ABBREVIATIONS

BMI: Body Mass Index
GWAS: Genome-Wide Association Study
IVW: Inverse Variance Weighted
MR: Mendelian randomization
PCOS: Polycystic Ovarian Syndrome
RCTs: Randomized Controlled Trials
SNP: Single Nucleotide Polymorphism
